# Comparison of the Gross Target Volumes Based on Diagnostic PET/CT for Primary Esophageal Cancer

**DOI:** 10.3389/fonc.2021.550100

**Published:** 2021-02-25

**Authors:** Jingzhen Shi, Jianbin Li, Fengxiang Li, Yingjie Zhang, Yanluan Guo, Wei Wang, Jinzhi Wang

**Affiliations:** ^1^ School of Medicine, Shandong University, Jinan, China; ^2^ Department of Radiation Oncology, Shandong Cancer Hospital Affiliated to Shandong University, Jinan, China; ^3^ Department of Radiation Oncology, Shandong Cancer Hospital and Institute, Shandong First Medical University and Shandong Academy of Medical Sciences, Jinan, China; ^4^ Department of Nuclear Medicine, Shandong Cancer Hospital and Institute, Shandong First Medical University and Shandong Academy of Medical Sciences, Jinan, China

**Keywords:** deformable image registration, three-dimensional computed tomography, ^18^F-FDG PET/CT, thoracic esophageal cancer, gross target volume

## Abstract

**Background:**

Clinically, many esophageal cancer patients who planned for radiation therapy have already undergone diagnostic Positron-emission tomography/computed tomography (PET/CT) imaging, but it remains unclear whether these imaging results can be used to delineate the gross target volume (GTV) of the primary tumor for thoracic esophageal cancer (EC).

**Methods:**

Seventy-two patients diagnosed with thoracic EC had undergone prior PET/CT for diagnosis and three-dimensional CT (3DCT) for simulation. The GTV_3D_ was contoured on the 3DCT image without referencing the PET/CT image. The GTV_PET-ref_ was contoured on the 3DCT image referencing the PET/CT image. The GTV_PET-reg_ was contoured on the deformed registration image derived from 3DCT and PET/CT. Differences in the position, volume, length, conformity index (CI), and degree of inclusion (DI) among the target volumes were determined.

**Results:**

The centroid distance in the three directions between two different GTVs showed no significant difference (*P* > 0.05). No significant difference was found among the groups in the tumor volume (*P* > 0.05). The median DI values of the GTV_PET-reg_ and GTV_PET-ref_ in the GTV_3D_ were 0.82 and 0.86, respectively (*P* = 0.006). The median CI values of the GTV_3D_ in the GTV_PET-reg_ and GTV_PET-ref_ were 0.68 and 0.72, respectively (*P* = 0.006).

**Conclusions:**

PET/CT can be used to optimize the definition of the target volume in EC. However, no significant difference was found between the GTVs delineated based on visual referencing or deformable registration whether using the volume or position. So, in the absence of planning PET–CT images, it is also feasible to delineate the GTV of primary thoracic EC with reference to the diagnostic PET–CT image.

## Introduction

According to the newly published GLOBOCAN 2018 study (1), esophageal cancer ranks seventh in cancer incidence and sixth in cancer mortality, with 572,000 new esophageal cancer cases and 590,000 deaths. Radiotherapy, as one of the main effective treatment modalities, is widely used in both the curative and palliative treatment of patients with EC (2–5). Modern radiotherapy techniques are largely affected by two crucial issues to accurately achieve tumor control: the precise quantification of tumor variations and complete identification of underlying tumor tissue (6, 7). Therefore, an increasing number of attempts have been made to accurately delineate and define the target volume.

Recently, the combination of multiple modalities has become one of the hottest topics in target determination research and plays a fundamental role in improving the accuracy of tumor delineation in EC. ^18^F-Fluorodeoxyglucose positron emission tomography/computed tomography (^18^F-FDG PET/CT), as a dual-modality imaging technique that provides both biological and metabolic information, has advantages in determining and correcting the gross tumor volume (GTV) as well as the extent of tumor motion in several directions (8, 9). Jin et al. (10) integrate the RTCT and PET modalities together into a two-stream chained deep fusion framework, which represents a complete workflow for the target delineation in esophageal cancer radiotherapy and pushes forward the state of automated esophageal GTV and CTV segmentation towards a clinically applicable solution. Using extensive five-fold cross-validation on 110 esophageal cancer patients, they also demonstrate that both the proposed two-stream chained segmentation pipeline that effectively fuses the CT and PET modalities *via* early and late 3D deep-network-based fusion and the PSNN model can significantly improve the accurate GTV segmentation over the previous state-of-the-art work (11). Yousefi S. et al. (12) found that the proposed method, dubbed dilated dense attention Unet (DDAUnet), could segment the gross tumor volume with a mean surface distance of 5.4 ± 20.2mm, demonstrating that a simplified clinical workflow based on CT alone could allow to automatically de-lineate the esophageal GTV with acceptable quality. Several studies also have demonstrated that adding PET data to radiation treatment planning (RTP) might significantly improve the accuracy of contouring tumors and reduce intra-observer and inter-observer variability in GTV delineation (13, 14). Moreover, PET–CT effectively assesses the responses to treatment and prognosis (15, 16).

Most patients with EC have had a diagnostic PET/CT scan before radiotherapy. Vesprini D. et al. (17) demonstrated that the addition of FDG-PET to computed tomography-based planning for the identification of primary tumor GTV in patients with gastro-esophageal carcinoma decreases both inter-observer and intra-observer variability. However, Nowee M. E. et al. (18) demonstrated that delineation variation of the primary tumor GTV can be considerable both on CT and on PET-CT fusion and is mainly located at the cranial and caudal border. Although the addition of FDG-PET to CT significantly impacted the delineated volume in two-third of the cases, PET did not translate into reduced observer variation at the cranial/caudal border in 50% of the patients with esophageal cancer. The delineation of the GTV only referencing diagnostic PET/CT leads to uncertainty for radiation oncologists. However, it is unlikely that these patients would undergo a second PET/CT scan owing to the significant cost and logistical problems involved, as well as the increased radiation exposure of the patient and staff during the scan. Thus, it is relatively difficult to popularize dedicated treatment planning PET/CT as routine management in clinical practice, and diagnostic PET/CT may be the only PET data provided for RTP. Hence, the feasibility of applying diagnostic PET–CT in delineating target volumes would contribute to the widespread application of diagnostic PET–CT in radiotherapy for EC.

Deformable image registration (DIR) is an image processing technique that maps voxels (the individual components) of a scan to those of another scan, striving to resolve differences in patient position and soft tissue displacement, and eventually generate accurately transferred and propagated volumetric tumor structures between image datasets (19, 20). Therefore, changes in the anatomical structure and position of patients between the planning CT and diagnostic PET/CT highlight the need for DIR in RTP. In several studies, the use of DIR has been demonstrated to permit the more accurate registration of diagnostic PET/CT scans to planning CT scans in patients with lung cancer and head and neck tumors (21–23). However, the clinical impact of DIR in target volume delineation after registering a diagnostic PET/CT scan on a planning CT scan for primary thoracic EC remains unclear.

Presently, planning CT remains the most widely used imaging modality to determine the GTV in clinical practice, although it is not the only standard imaging approach. Therefore, the present study aimed to evaluate geometrical differences in the GTV contoured on planning CT, referencing PET/CT and the GTV contoured on the deformed image derived from planning CT and PET/CT for primary thoracic EC.

## Methods and Materials

### Patient Selection and Characteristics

Our institute research ethics board approved this study, and informed consent was provided by each patient before enrollment in the study. Seventy-two patients with pathologically proven EC and planned for radiotherapy at our hospital were consecutively enrolled between July 2013 and July 2018. None of the patients were scheduled to accept radiotherapy or chemotherapy previously. The 72 patients had undergone diagnostic PET/CT before treatment not longer than a week. Patients with an absolute maximal standardized uptake value (SUVmax) ≥2 were enrolled, and the delineation standard chosen in our study was the absolute SUVmax ≥2.5. In total, the imaging data from 72 patients were available for analysis in our study. The patient characteristics are listed in **Table 1**.

**Table 1:** Characteristics of patients enrolled in the study.

Parameters	Parameters
Sex	
Male	64
Female	8
Age, median, years (range)	44–88 (63)
Tumor location	
Upper	32
Middle	24
Distal	16
SUV_max_	3.21–49.50 (mean: 12.95)
Pathological type	
Squamous	70
Adenocarcinoma	2

### Deformable Image Registration of PET/CT Scan to Planning CT Scan

**Figure 1:**
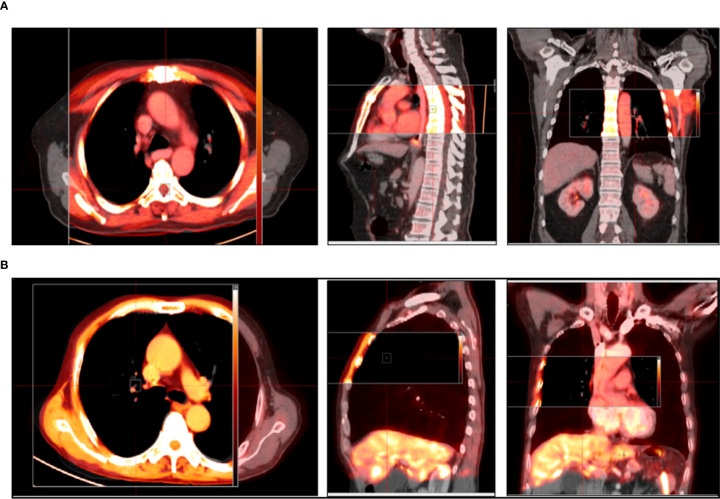
**(A, B)** Representative images in transversal, sagittal, and coronal sections show an ideal match between the before images and after images using DIR. The gray images represent images before deformable registration, and the red images represent images after deformable registration.

DIR at our institution was performed using MIM software, an intensity-based, free-form, deformable registration algorithm with limitless degrees of freedom. This algorithm has been evaluated for clinical use by Piper (24). Given the low resolution of PET images and lack of clear discernible normal landmarks, in all cases, registration was performed using the CT components of the PET/CT and planning CT scans. Resampling of the PET scan was performed using the registration results from the deformable CT–CT registration. For CT–CT registration, initially, a rigid registration focusing on the dorsal spine was performed automatically using MIM software. Following rigid registration, deformable registration was then performed. In this process, the CT component of the PET/CT scan was deformed to the planning CT scan using the deformable registration algorithm described above. Finally, we placed a bounding box over the region of interest (ROI) to remove the influence of other parts of the body because the automatic image registration algorithm considers the entire image dataset. At the same time, PET voxels were mapped to the planning CT scan during the transformation used in the CT–CT registration process, resulting in a new PET/CT dataset that was deformably registered to the planning CT scan. Novel frameworks for deformable registration evaluation and quality assurance were provided by MIM software to inspect different properties of a deformable registration between two volumes. After deformable registration was completed, we first tested the intensity-based free-form deformable registration algorithm using detailed statistics obtained from evaluation tools, such as the Hausdroff Distance, Jaccard Coefficient, Dice Score, and Standard Deviation. Finally, an experienced radiotherapist performed visual assessment using Reg Reveal, an evaluation tool provided by MIM software, and then manual modification to ensure adequate accuracy of deformable registration. In our study, we also provided the deformed images of two patients to demonstrate the accuracy of DIR in MIM software (**Figures 1A, B**).

### Target Volume Delineation

**Figure 2:**
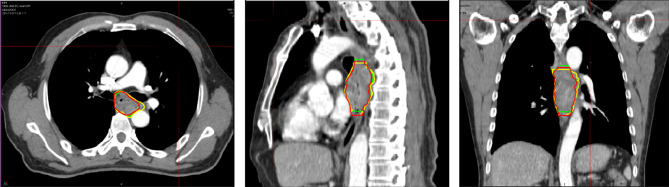
Representative images contoured in the image sessions in the transversal (a1), sagittal (a2), and coronal sections (a3) show differences among the three target volume delineations. The yellow contour represents the GTV_3D_, the red contour represents the GTV_PET-ref_, and the green contour represents the GTV_PET-reg._ GTV, gross target volume; GTV3D, GTV contoured based on 3DCT without referencing 18F-FDG PET/CT; GTVPET-ref, GTV contoured on 3DCT referencing 18F-FDG PET/CT; GTVPET-reg, GTV contoured on the deformed image derived from 3DCT and 18F-FDG PET/CT using MIM deformable registration software.

All target volumes were contoured using MIM software. Although a total of six doctors participated in the course of GTV definition in this study, but in fact, all patients’ GTVs were delineated by the same radiotherapist. The other five doctors were respectively responsible for the development of GTV delineation standards and review of the delineated GTVs. First of all, the delineated standards were together developed by two experienced radiation oncologists and one experienced imaging specialist before performing our study. Using the mediastinal window settings (window width = 400 HU, window level = 40 HU), GTV_S_ were delineated following the standards for an esophageal wall thickness >5 mm or an esophageal wall diameter (without gas) >10 mm. Our study only evaluated the primary tumor; however, if positive lymph nodes could not be separated from the primary tumor visually, they were delineated together with the primary tumor. First, GTV_3D_ was manually contoured based on planning CT by an experimented radiotherapist who did not know the diagnostic PET/CT results. After two weeks, regarding the planning CT scan as the primary image and referencing the high-metabolism region observed on PET–CT, the same radiotherapist added the high-metabolism region observed visually on PET/CT without planning CT and removed the low-metabolism region observed visually on PET/CT within planning CT. In a word, the uncertainty or excludable region in CT images could be determined by referencing PET–CT images. The new GTV, resulting from referencing PET/CT, was referred to as GTV_PET-ref_. Two weeks later, the GTV was first automatically contoured on the new deformed image derived from planning CT and PET/CT using SUV values by MIM software and then manually modified taking the registered PET–CT as reference by the same radiotherapist. Finally, the contour was named GTV_PET-reg_. During the auto-contouring process, we first position the sphere over the region of interest and resize the sphere with a right-click drag up or down from inside the sphere, then setting the exact threshold by clicking on the threshold and typing. Finally, contour was generated by clicking the green Checkmark Button at the right edge of the viewport. Based on many previous studies investigating the optimal method of PET-based target volume delineation, the PET-based delineation method of an absolute SUV threshold of 2.5 and a maximum standardized uptake value (SUVmax) threshold of 20% were used in the automatically contoured target images by MIM software in our study (25–27). It should be make clear that the absolute SUVs of the heart were less than two for most of the patients in our study, and we would repair the heart using CT image for some cases with a SUV≥2. The mediastinal high-metabolism region was first contoured based on PET–CT, and then the boundary of the heart was determined using CT images. Finally, the heart would be repaired referencing the high-metabolism region observed on PET–CT and the heart border observed on CT images. The observer strictly adhered to the standards and was guided by two experienced radiation oncologists throughout the delineation. After all GTVs were delineated, another radiotherapist and a nuclear medicine doctor reviewed the images again. In our study, we also provided pictures of one patient to show differences among the three target volume delineations (**Figure 2**).

### GTV Comparison

Any two GTVs were compared in terms of the position, volume, length, conformity CI, and DI.

To assess the positional change, the center of mass (COM) coordinates for the GTV_3D_, GTV_PET-ref_, and GTV_PET-reg_ for each patient were measured. Next, the displacement in the x (left–right, LR), y (anterior–posterior, AP) and z (cranial–caudal, CC) directions between two different GTVs was obtained as ≥x, ≥y, and ≥z, respectively. The 3D vector was calculated according to the following formula: V = (≥x^2^ + ≥y^2^ +≥z^2^)^1/2^.

The CI (28) and DI (29) were determined to assess changes in volumetric shape and position. The CI of volumes A and B [CI (A, B)] was defined as the ratio of the intersection of A with B to the union of A and B; that is, CI = A∩B/A∪B. The ideal value of CI is 1 if the two volumes are identical. With any change in the size, position, shape, or orientation, the value of CI would be less than 1. The definition of the DI of volume A included in B [DI (A in B)] is the intersection between volume A and B in volume A. The formula is as follows: DI (A in B) = A∩B/A. Assuming volume B is the reference for the standard volume, if treatment planning is based on volume A, there will be 1-DI (A in B) of volume A being unnecessarily irradiated and 1-DI (A in B) of volume B missing irradiation.

### Statistical Analysis

Statistical analysis was performed using the SPSS software package (SPSS 22.0). The Wilcoxon test was used to compare the position, volume, CI, and DI, and paired sample t test was used to examine differences in the maximum transverse diameter and tumor length between target volumes. The *Z* values represent the test statistics. Values of *P <*0.05 were regarded as significant. The degree of association between GTV motion vectors and continuous variables (such as the CI) was calculated by the Spearman test.

## Results

To investigate the correlation between different locations of EC and changes in position, the patients were divided into three groups according to the 2007 Tumor Node Metastasis (TNM) classification system of the National Comprehensive Cancer Network (NCCN): group A: 32 patients with lesions located in the proximal segment; group B: 24 patients with lesions located in the middle segment; group C: 16 patients with lesions located in the distal segment.

### Difference in the Tumor Centroid Distance

**Table 2** shows the differences in the position between GTV_PET-reg_ and GTV_PET-ref_, GTV_PET-reg_ and GTV_3D_, and GTV_PET-ref_ and GTV_3D_. The centroid distance in the three directions between two different GTVs showed no significant difference in any patient (*P* > 0.05). However, differences were found between GTV_PET-reg_ and GTV_3D_ (*Z* = 4.94; *P* = 0.000), as well as between GTV_PET-ref_ and GTV_3D_ (*Z* = −4.94; *P* = 0.000) in group A.

**Table 2:** Displacement of the 3D vector between different gross target volumes for all and different segments of patients (*X ± S*, mm).

Group	Total (n = 72)	Group A(n = 32)	Group B(n = 24)	Group C(n = 16)
GTV_PET-reg_ and GTV_PET-ref_	2.54 ± 7.41	0.80 ± 1.72	1.61 ± 2.70	5.65 ± 14.06
GTV_PET-reg_ and GTV_3D_	1.32 ± 2.40	0.93 ± 1.70	1.62 ± 2.70	4.19 ± 13.32
GTV_PET-ref_ and GTV_3D_	1.92 ± 7.00	0.64 ± 0.52	1.82 ± 0.99	4.96 ± 13.98
*Z,P*	−0.23,0.815	−0.49,0.620	0.32, 0.753	0.65, 0.529
	−0.06,0.946	4.94,0.000	−4.29,0.068	−0.47,0.638
	−0.11,0.912	−4.94,0.000	−7.56,0.125	0.03, 0.975

3D, represents the centroid distance in the 3D directions between two different GTVs; GTV, gross target volume; LR, left–right; AP, anterior–posterior; CC, cranial–caudal; GTV_3D_, GTV contoured based on 3DCT without referencing ^18^F-FDG PET/CT; GTV_PET-ref_, GTV contoured on 3DCT referencing ^18^F-FDG PET/CT; GTV_PET-reg_, GTV contoured on the deformed image derived from 3DCT and ^18^F-FDG PET/CT using MIM deformable registration software.

### Differences in the Tumor Volume

**Table 3** shows tumor volumes for all patients and the three groups of patients. In group C, the GTV_3D_ was significantly less than the GTV_PET-ref_ (*Z* = 2.430; *P* = 0.015), and no significant difference was found between GTV_PET-reg_ and GTV_PET-ref_ (*Z* = −1.823; *P* = 0.068) or between GTV_PET-reg_ and GTV_3D_ (*Z* = 0.402; *P* = 0.687).

**Table 3:** Target volumes of the GTV_3D_, GTV_PET-ref_, and GTV_PET-reg_ for patients in group C (n = 16).

GTV(cm^3^)	M	IQR	X ± S
GTV_3D_	44.82	(19.76, 55.47)	48.65 ± 40.11
GTV_PET-ref_	49.87	(28.93, 55.56)	55.75 ± 41.18
GTV_PET-reg_	41.15	(31.66, 41.15)	53.29 ± 36.82

M, median; IQR, interquartile range; GTV, gross target volume; GTV_3D_, GTV contoured based on 3DCT without referencing ^18^F-FDG PET/CT; GTV_PET-ref_, GTV contoured on 3DCT referencing ^18^F-FDG PET/CT; GTV_PET-reg_, GTV contoured on the deformed image derived from 3DCT and ^18^F-FDG PET/CT by MIM deformable registration software.

### Differences in the Tumor Length and Maximum Transverse Diameter

**Table 4** shows the lengths and maximum transverse diameters of the GTVs. The paired sample t test results indicated that the length of the GTV_PET-ref_ was significantly longer than that of the GTV_3D_ in entire group patients, and patients in group C (*t* = 2.134, 3.204; *P* = 0.033,0.001).

**Table 4:** Maximum transverse diameter of the Target volumes and tumor length Target volumes of the GTV_3D_, GTV_PET-ref_, and GTV_PET-reg_ for all and different segments of patients (*X ± S*).

Group	Length (cm)			Maximum transverse diameter (cm)		
	GTV_3D_	GTV_PET-ref_	GTV_PET-reg_	GTV_3D_	GTV_PET-ref_	GTV_PET-reg_
Total (n = 72)	8.54 ± 3.43	9.29 ± 4.48	8.38 ± 4.21	3.94 ± 1.00	3.75 ± 0.94	3.80 ± 0.94
Group A(n = 32)	8.41 ± 3.57	8.56 ± 4.99	7.50 ± 3.91	3.62 ± 0.82	3.49 ± 0.76	3.49 ± 0.73
Group B(n = 24)	8.52 ± 2.54	9.51 ± 3.78	8.71 ± 3.39	4.05 ± 1.00	3.76 ± 0.89	3.89 ± 0.96
Group C(n = 16)	8.58 ± 4.44	10.30 ± 4.59	9.53 ± 5.18	4.15 ± 1.09	4.02 ± 1.11	3.87 ± 1.07

GTV, gross target volume; GTV_3D_, GTV contoured based on 3DCT without referencing ^18^F-FDG PET/CT; GTV_PET-ref_, GTV contoured on 3DCT referencing ^18^F-FDG PET/CT; GTV_PET-reg_, contoured on the deformed image derived from 3DCT and ^18^F-FDG PET/CT using MIM deformable registration software.

The paired sample t test results indicated that the maximum transverse diameters of the GTV_PET-reg_ and GTV_PET-ref_ were less than that of the GTV_3D_ for entire group patients, patients in group A and patients in group B (*t* = −3.891–3.716; *P* < 0.05). However, no significant difference was observed in group C (*t* = 0.778–1.678; *P* = 0.449–0.114).

### Differences in the CI and DI

**Table 5** illustrates the CIs for all patients with EC. The median CI between the GTV_PET-reg_ and GTV_3D_ was less than that between the GTV_PET-ref_ and GTV_3D_ (*Z* = −2.756; *P* = 0.006). The median CI between the GTV_PET-reg_ and GTV_3D_ was less than that between the GTV_PET-reg_ and GTV _PET-ref_ (*Z* = −2.244; *P* = 0.025). Furthermore, the CI between the GTV_PET-reg_ and GTV_PET-ref_, GTV_PET-ref_ and GTV_3D_, and GTV_PET-reg_ and GTV_3D_ showed a significant negative correlation with the 3D vector for all patients (R = −0.344, −0.517, −0.527; *P* < 0.05).

**Table 5:** CI between different gross target volumes for all patients (n = 72).

CI	M	IQR	X ± S
GTV_PET-reg_ and GTV_PET-ref_	0.69	(0.62, 0.78)	0.67 ± 0.15
GTV_PET-reg_ and GTV_3D_	0.68	(0.56, 0.78)	0.64 ± 0.19
GTV_PET-ref_ and GTV_3D_	0.72	(0.57, 0.78)	0.68 ± 0.15

CI, conformity index; M, median; IQR, interquartile range; GTV, gross target volume; GTV_3D_, GTV contoured based on 3DCT without referencing ^18^F-FDG PET/CT; GTV_PET-ref_, GTV contoured on 3DCT referencing ^18^F-FDG PET/CT; GTV_PET-reg_, GTV contoured on the deformed image derived from 3DCT and ^18^F-FDG PET/CT using MIM deformable registration software for all patients.

The DIs for all patients and the three groups are shown in **Table 6**. The median DI of the GTV_PET-reg_ in GTV_3D_ was less than that of the GTV_PET-ref_ in GTV_3D_ (*Z* = −2.741; *P* = 0.006). However, the difference in the DI between the GTV_3D_ in GTV_PET-reg_ and GTV_3D_ in GTV_PET-ref_ was not significant (*Z* = 1.429; *P* = 0.131). In group C, the median DI of the GTV_PET-reg_ in GTV_3D_ was less than that of the GTV_PET-ref_ in GTV_3D_ (*Z* = −2.534; *P* = 0.001). Furthermore, the DI of GTV_3D_ in GTV_PET-reg_ was less than that of the GTV_3D_ in GTV_PET-ref_ (*Z* = 2.275, *P* = 0.023). Additionally, the DI of GTV_PET-reg_ in GTV_3D_ was less than that of the GTV_PET-reg_ in GTV_PET-ref_ (*Z* = 2.585; *P* = 0.010). However, a significant difference in the DI was not found between the GTV_PET-ref_ in GTV_PET-reg_ and GTV_3D_ in GTV_PET-reg_ (*Z* = 0.052; *P* = 0.959).

**Table 6:** DIs of different gross target volumes for all and different segments of patients.

DI	Total (n=72)		Group A(n=32)		Group B(n=24)		Group C(n=16)	
	M,IQR	X ± S	M,IQR	X ± S	M,IQR	X ± S	M,IQR	X ± S
GTV_PET-reg_ **in** GTV_3D_	0.82 (0.75,0.90)	0.78 ± 0.20	0.84 (0.75,0.90)	0.82 ± 0.13	0.82(0.78,0.90)	0.82 ± 0.10	0.76(0.32,0.90)	0.60 ± 0.35
GTV_PET-ref_ **in** GTV_3D_	0.86 (0.76,0.92)	0.81 ± 0.19	0.87 (0.78,0.92)	0.84 ± 0.13	0.84(0.77,0.92)	0.83 ± 0.11	0.83(0.40,0.90)	0.70 ± 0.25
GTV_3D_ **in** GTV_PET-reg_	0.84 (0.66,0.92)	0.77 ± 0.22	0.82 (0.68,0.92)	0.80 ± 0.14	0.85(0.65,0.93)	0.78 ± 0.20	0.83(0.40,0.90)	0.65 ± 0.40
GTV_3D_ **in** GTV_PET-ref_	0.87 (0.72,0.93)	0.81 ± 0.15	0.82 (0.72,0.92)	0.81 ± 0.15	0.86(0.70,0.92)	0.80 ± 0.15	0.90(0.81,0.95)	0.83 ± 0.18
GTV_PET-reg_ **in** GTV_PET-ref_	0.82 (0.76,0.89)	0.80 ± 0.13	0.84 (0.78,0.89)	0.82 ± 0.10	0.80(0.74, 0.89)	0.80 ± 0.12	0.83(0.67,0.90)	0.76 ± 0.22
GTV_PET-ref_ **in** GTV_PET-reg_	0.84 (0.77,0.90)	0.81 ± 0.15	0.84 (0.78,0.91)	0.83 ± 0.10	0.85(0.80, 0.89)	0.80 ± 0.20	0.80(0.65,0.88)	0.75 ± 0.17

DI, degree of inclusion; M, median; IQR, interquartile range; GTV, gross target volume; GTV_3D_, GTV contoured based on 3DCT without referencing ^18^F-FDG PET/CT; GTV_PET-ref_, GTV contoured on 3DCT referencing ^18^F-FDG PET/CT; GTV_PET-reg_, GTV contoured on the deformed image derived from 3DCT and ^18^F-FDG PET/CT using MIM deformable registration software.

## Discussion

PET/CT-guided precise radiotherapy for EC is now widely accepted by radiologists. Most patients with EC have already undergone diagnostic PET/CT imaging before radiotherapy simulation. Thus, how to delineate and define the target volume using diagnostic PET/CT efficiently, economically, and simply is an urgent issue. Because of the lack of validation, unfortunately, the volume contour of EC only referencing PET/CT, which is widely used clinically, may lead to uncertainty for radiation oncologists.

Incorporating diagnostic PET/CT into planning CT using rigid image registration (RIR) may lead to misalignment and cannot be used clinically because of significant changes in patient position and anatomy. DIR may be a powerful tool that potentially account for such changes by estimating the non-uniform or non-linear relationships between the volumetric elements of corresponding structures in the imaging data (30). However, DIR is also time-saving for clinicians and reduces the intra-observer variability by automatically defining the target volumes with a determined SUV-based thresholding strategy (31). The performance and utility of DIR have been investigated to allow for its application in patients with head and neck tumors (32–34).

However, few studies have aimed to improve the accuracy of the target volume definition for primary thoracic EC in recent years (35, 36), and related research has only explored the advantages of deformable registration.

We initially analyzed the tumor position variation in different directions for all subjects with EC and found a significant difference in the displacement of the COM in the AP direction between the GTV_PET-ref_ or GTV_3D_ and GTV_PET-reg_ (*P* = 0.037, 0.000). However, no significant difference was observed in the displacement in the LR or CC direction in any comparison between two different GTVs (*P* = 0.178−0.771). Furthermore, we compared the position variation in all three groups, and minimal variation was found for tumors in the middle lobe, with no significant difference in the LR, AP or CC direction (*P* = 0.127−0.550). By contrast, patients with distal EC showed the maximal variation, with a difference in the centroid coordinates between GTV_PET-reg_ and GTV_PET-ref_ (*P* = 0.031) or GTV_3D_ (*P* = 0.021) in the AP direction. In patients with upper lobe tumors, a significant difference was found in the centroid coordinates between GTV_PET-reg_ and GTV_3D_ in the AP direction (*P* = 0.017). The magnitude of motion was larger for tumors in the distal lobe than tumors in the upper and middle lobes due to peristalsis, respiration, and involuntary motion. Additionally, anatomical structures around the distal esophagus are complex. Therefore, these observations may suggest that tumor motion and its adjacent structures are still key factors leading to target position variation between all contours based on planning CT scans, planning CT scans referencing PET/CT scans and planning CT scans registered using DIR to PET/CT scans. The findings also suggest that differences in the LR and CC directions should be noted when using PET/CT for EC. Thus, no significant measurements based on PET/CT, either deformed or referenced, should be performed in these two directions.

Thoracic EC targets are generally cylinder-like in contour with a long vertical axis and a short horizontal axis. The tumor length and maximum transverse diameter are key factors influencing the GTV. Hence, measurement of the GTV based on different imaging modalities, which not only can reflect the tumor shape but also indicate volumetric changes, would be beneficial to our choice of appropriate images to construct the target volume. When comparing the tumor length, we found that the length of the GTV_PET-ref_ was significantly longer than that of the GTV_3D_ for all the patients with EC and those in group C (distal EC) (*P* = 0.033; *P* = 0.001). However, a significant result was not observed between the GTV_PET-reg_ and GTV_PET-ref_ or GTV_3D_ for all patients or patients in the three groups. Thus, no difference was found in the length between the GTV contoured on planning CT registered using DIR with PET/CT and that contoured on planning CT referencing PET/CT, although the latter showed a trend of increasing longitudinal length. Hong et al. (37) reported that the mean length of PET/CT-based contours (6.53 cm) was longer than that of CT-based contours (4.8 cm). Additionally, Grange et al. (38) compared the GTVs derived from 3DCT images and PET–CT images and demonstrated that the GTV_PET_ (12.6 cm) was longer than the GTV_3D_ (11.7 cm). Furthermore, we analyzed the maximum transverse tumor diameter and found that the maximum transverse diameters of the GTV_PET-reg_ and GTV_PET-ref_ were less than that of the GTV_3D_ in all patients, patients in group A (upper EC) and patients in group B (middle EC); however, no significant difference was observed between the GTV_PET-reg_ and GTV_PET-ref_. The upper and middle thoracic esophagus are adjoined to high-density tissues, such as the trachea and cardiac tissue, while the distal thoracic esophagus is mainly adjoined to the lungs, which are low-density organs. Therefore, our study reflects the advantage of PET/CT in distinguishing tumors from high-density tissues, and this advantage is not affected by deriving the GTV from 3DCT referencing PET/CT or deformed PET/CT. We also compared the GTV based on the length and maximum transverse diameter of the tumor. Our study showed that the volume of the GTV_PET-ref_ was significantly greater than that of the GTV_3D_ in group C, and the difference between GTV_PET-ref_ and GTV_PET-reg_ was close to statistical significance (*P* = 0.068). These results reveal that the distal esophagus will be most influenced by the tumor length among all three segments.

The CI and DI reflect synthetically geometrical differences in the two selected target volumes and can be affected by factors such as the volumetric shape, size, and spatial position. Our study showed that the median CIs approximated to 0.7—that is, any two different GTVs among GTV_PET-reg_, GTV_PET-ref_ and GTV_3D_ corresponded well. However, the CIs of the GTV_PET-reg_ and GTV_3D_ were significantly lower than those of the GTV_PET-ref_ and GTV_3D_ (*P* = 0.006), and the CIs of the GTV_PET-reg_ and GTV_PET-ref_ showed a significant inverse correlation with the centroid distance (r = −0.517; *P* < 0.05). These results indicate that the GTVs contoured on deformed images derived from 3DCT and PET/CT correlated well with those contoured on 3DCT referencing PET/CT. With increasing centroid distance between the GTV_PET-reg_ and GTV_PET-ref_, the conformity decreased significantly. However, compared with the GTVs contoured on deformed images derived from 3DCT and PET/CT, the GTVs contoured on 3DCT referencing PET/CT corresponded better with the GTVs contoured on 3DCT. The cause may be the automatic delineation performed by MIM software, which uses an absolute SUV threshold of 2.5 and an SUV_max_ threshold of 20% within the target image, leading to changes in the shape and size of the tumor. By further analyzing the DI between the GTV_PET-reg_ or GTV_PET-ref_ and GTV_3D_, we found that the difference in the DI between the GTV_PET-reg_ in GTV_3D_ (0.82) and that of the GTV_PET-ref_ in GTV_3D_ (0.86) was also statistically significant (*Z* = −2.741; *P* = 0.006). Although a GTV contoured on planning CT is not a standard volume, it is still often referred to as a control target volume due to its wide clinical application. In our study, the GTVs contoured on 3DCT referencing PET/CT correlated better with those contoured on 3DCT in terms of both the CI and DI compared with GTVs contoured on deformed images derived from 3DCT and PET/CT. These results indicate that using the GTV_PET-reg_ for RTP will increase the unnecessary irradiation of normal tissues and the amount of unirradiated tumor tissue. Therefore, we suggest that caution should be exercised when applying DIR to propagate target volumes between planning CT and diagnostic PET/CT for EC; further research is needed before large-scale clinical applications. Our results are consistent with those of a previous study by Guo et al. at our institution (25). They also proved that the clinical application of DIR between diagnostic PET/CT and planning CT should be performed with caution. Hanna et al. (13) performed DIR between diagnostic PET/CT and planning CT for 10 patients with lung cancer; the results suggested that deformable registration had no obvious advantage in defining the target volume due to inconsistent CI results between PET-based and CT-based contours.

Notably, none of the thresholding approaches have been standardized to conduct PET/CT-based delineation. Thus, the accuracy of tumor delineation in EC may be affected by different SUV-based thresholding strategies, leading to the misjudgment of high-intake regions, resulting in sites of necrosis and esophagitis being included as tumor tissues. Additionally, our study was conducted under the premise that PET imaging in PET–CT exactly matches CT imaging in PET–CT; however, PET scans containing several breathing cycles provide functional information, and CT scans obtain images rapidly in only a certain position in a specific single phase, possibly resulting in a mismatch due to respiratory motion (35). Of course, it cannot be denied that the inter-observer variability are un-avoidable for esophageal GTV delineation even using PET-CT (17, 18), and there has been no report on the difference between the GTVs with reference to the diagnostic PET–CT image by the different observers. So, we will ask one of those five doctors to delineate some of the patients and evaluate his/her performance under those three conditions to study this difference in future.

## Conclusion

Although some differences were observed between the GTV_PET-reg_ and GTV_PET-ref_ for distal EC regions with significant anatomical changes, no significant difference was found among all patients with EC between the GTVs contoured on 3DCT referencing diagnostic PET/CT and GTVs contoured on deformed images derived from 3DCT and diagnostic PET/CT in either the volume or spatial position. The CI and DI of the GTV_PET-ref_ and GTV_3D_ were better than those of the GTV_PET-reg_ and GTV_3D_. Therefore, PET/CT can be used to optimize the definition of the target volume in EC. However, no significant difference was found between the GTVs delineated based on visual referencing or deformable registration whether using the volume or position. So, in the absence of planning PET–CT images, it is also feasible to delineate the GTV of primary thoracic EC with reference to the diagnostic PET–CT image.

## Data Availability Statement

The raw data supporting the conclusions of this article will be made available by the authors, without undue reservation.

## Ethics Statement

The studies involving human participants were reviewed and approved by Shandong Cancer Hospital and Institute. The patients/participants provided their written informed consent to participate in this study. Written informed consent was obtained from the individual(s) for the publication of any potentially identifiable images or data included in this article.

## Author Contributions

FL, JL, and JS conceived and designed the study and drafted the manuscript. FL and JL provided administrative support. JS and YZ contributed to the data collection and assembly. JS and YG participated in analyzing and interpreting the data. WW and JW participated in revising the content. All authors contributed to the article and approved the submitted version.

## Funding

This study was supported by the Key Research Development Program of China (201 6YFC0904700), National Natural Science Foundation of China (81773287), and Natural Science Foundation of Shandong Province (ZR2019PH115).

## Conflict of Interest

The authors declare that the research was conducted in the absence of any commercial or financial relationships that could be construed as a potential conflict of interest.

## Appendix

### CT Image Acquisition

The X-ray image acquisition protocol was signed by every patient before CT scanning. Two localization methods are used for patients with EC in clinical practice, one using a negative pressure band with the arms raised over the head and the other using a thermoplastic mask with the arms placed along the side of the body. Patients with lower thoracic esophageal cancer would choose to use negative pressure bands, and patients with EC requiring irradiation of the neck and upper mediastinum always choose to use thermoplastic masks. Furthermore, using a thermoplastic mask would also reduce some changes caused in the position by keeping the shoulders fixed. Therefore, to unify research standards, all the patients in our study were immobilized using a thermoplastic mask in the supine position with the arms placed along the side of the body during the simulation. Three laser alignment markers were placed on the patient before image acquisition. Each patient was injected with 85 ml of contrast medium and rested for approximately 45 s. Next, enhanced planning CT of the thoracic region was performed using a 16-slice CT scanner (Philips Brilliance Bores CT, Cleveland, OH, USA) under uncoached free-breathing conditions for every patient. Planning CT was performed at a thickness of 2.4 mm per gantry rotation (1 s) and interval (1.8 s) between rotations. The three-dimensional computed tomography (3DCT) scanning procedure requires approximately 30 s. The planning CT images were reconstructed using a thickness of 3 mm and then were transferred to an Eclipse treatment planning system (Eclipse 15.5; Varian Medical Systems, Palo Alto, USA).

### PET/CT Image Acquisition

PET–CT was performed no longer than a week before the planning CT as part of the routine diagnostic management for EC. All the patients were asked to fast for at least 6 before the PET/CT examination. Each patient was injected with 7.4 MBq/kg body weight of ^18^F-FDG and then rested for approximately 1 in a quiet room before scanning. ^18^F-FDG PET/CT scan of the chest was performed using an integrated PET/CT scanner (Philips Gemini TF, Cleveland, OH, USA). The patients were positioned on a conventional curved diagnostic bed with a pillow for head support. The 16-slice CT component was operated at a peak X-ray tube voltage of 140 kV, 90 mA, a slice thickness of 5 mm and an interval of 4 mm and was used for both PET data attenuation correction and ^18^F-FDG uptake localization in PET images. CT images without contrast were obtained during free breathing. A dedicated PET scan was performed covering the same axial range for 2 min per bed position (a total of 6–7 bed positions). The PET images were reconstructed using CT-derived attenuation correction and an ordered subset expectation maximization algorithm with a postreconstruction Gaussian filter at 5 mm full width at half maximum and then were transferred to MIM software (MIM 6.8.5; MIM Software Inc., Cleveland, OH, USA).
